# A systematic review and meta-analysis of diagnostic performance comparison between generative AI and physicians

**DOI:** 10.1038/s41746-025-01543-z

**Published:** 2025-03-22

**Authors:** Hirotaka Takita, Daijiro Kabata, Shannon L. Walston, Hiroyuki Tatekawa, Kenichi Saito, Yasushi Tsujimoto, Yukio Miki, Daiju Ueda

**Affiliations:** 1 Department of Diagnostic and Interventional Radiology, Graduate School of Medicine, Osaka Metropolitan University, Osaka, Japan; 2 Center for Mathematical and Data Science, Kobe University, Kobe, Japan; 3 Department of Artificial Intelligence, Graduate School of Medicine, Osaka Metropolitan University, Osaka, Japan; 4 Center for Digital Transformation of Health Care, Graduate School of Medicine, Kyoto University, Kyoto, Japan; 5 Oku Medical Clinic, Osaka, Japan; 6 Department of Health Promotion and Human Behavior, Kyoto University Graduate School of Medicine/School of Public Health, Kyoto University, Kyoto, Japan; 7 Scientific Research WorkS Peer Support Group (SRWS-PSG), Osaka, Japan; 8 Center for Health Science Innovation, Osaka Metropolitan University, Osaka, Japan

**Keywords:** Medical research, Diagnosis

## Abstract

While generative artificial intelligence (AI) has shown potential in medical diagnostics, comprehensive evaluation of its diagnostic performance and comparison with physicians has not been extensively explored. We conducted a systematic review and meta-analysis of studies validating generative AI models for diagnostic tasks published between June 2018 and June 2024. Analysis of 83 studies revealed an overall diagnostic accuracy of 52.1%. No significant performance difference was found between AI models and physicians overall (*p* = 0.10) or non-expert physicians (*p* = 0.93). However, AI models performed significantly worse than expert physicians (*p* = 0.007). Several models demonstrated slightly higher performance compared to non-experts, although the differences were not significant. Generative AI demonstrates promising diagnostic capabilities with accuracy varying by model. Although it has not yet achieved expert-level reliability, these findings suggest potential for enhancing healthcare delivery and medical education when implemented with appropriate understanding of its limitations.

## Introduction

In recent years, the advent of generative artificial intelligence (AI) has marked a transformative era in our society^1–8^. These advanced computational systems have demonstrated exceptional proficiency in interpreting and generating human language, thereby setting new benchmarks in AI’s capabilities. Generative AI, with its deep learning architectures, has rapidly evolved, showcasing a remarkable understanding of complex language structures, contexts, and even images. This evolution has not only expanded the horizons of AI but also opened new possibilities in various fields, including healthcare^9^.

The integration of generative AI models in the medical domain has spurred a growing body of research focusing on their diagnostic capabilities^10^. Studies have extensively examined the performance of these models in interpreting clinical data, understanding patient histories, and even suggesting possible diagnoses^11,12^. In medical diagnosis, the accuracy, speed, and efficiency of generative AI models in processing vast amounts of medical literature and patient information have been highlighted, positioning them as valuable tools. This research has begun to outline the strengths and limitations of generative AI models in diagnostic tasks in healthcare.

Despite the growing research on generative AI models in medical diagnostics, there remains a significant gap in the literature: a comprehensive meta-analysis of the diagnostic capabilities of the models, followed by a comparison of their performance with that of physicians. Such a comparison is crucial for understanding the practical implications and effectiveness of generative AI models in real-world medical settings. While individual studies have provided insights into the capabilities of generative AI models^13,14^, a systematic review and meta-analysis are necessary to aggregate these findings and draw more robust conclusions about their comparative effectiveness against traditional diagnostic practices by physicians.

This paper aims to bridge the existing gap in the literature by conducting a meticulous meta-analysis of the diagnostic capabilities of generative AI models in healthcare. Our focus is to provide a comprehensive diagnostic performance evaluation of generative AI models and compare their diagnostic performance with that of physicians. By synthesizing the findings from various studies, we endeavor to offer a nuanced understanding of the effectiveness, potential, and limitations of generative AI models in medical diagnostics. This analysis is intended to serve as a foundational reference for future research and practical applications in the field, ultimately contributing to the advancement of AI-assisted diagnostics in healthcare.

## Results

### Study selection and characteristics

**Fig. 1:**
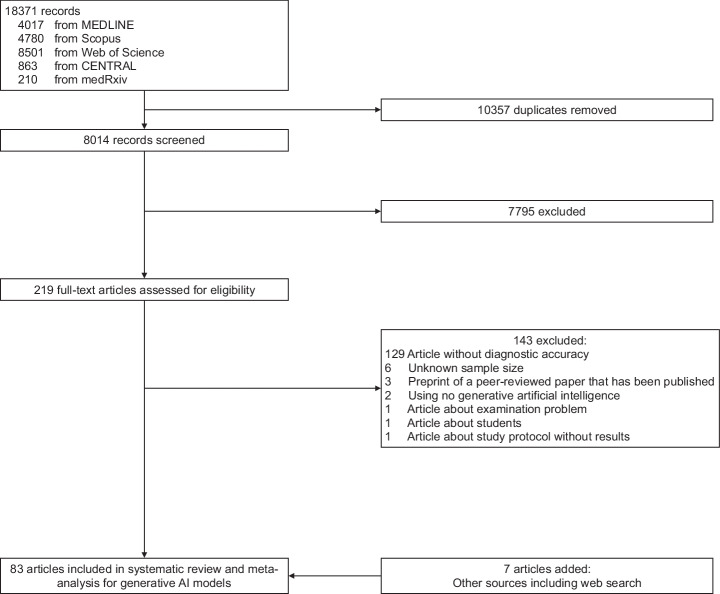
Eligibility criteria. The flow diagram illustrates the systematic review process, starting with 18,371 initial records identified from multiple databases: 4017 from MEDLINE, 4780 from Scopus, 8501 from Web of Science, 863 from CENTRAL, and 210 from medRxiv. After removing 10,357 duplicates, 8014 records were screened. Of these, 7795 were excluded as they did not align with the objectives of this systematic review, leaving 219 full-text articles for eligibility assessment. Further evaluation resulted in 143 exclusions due to various reasons: 129 articles without diagnostic accuracy, 6 with unknown sample size, 3 preprints of already published peer-reviewed papers, 2 not using generative artificial intelligence, 1 article about examination problems, 1 about students, and 1 about study protocol without results. Seven additional articles were identified through other sources including web search, resulting in a final total of 83 articles included in the systematic review and meta-analysis focusing on generative AI models.

We identified 18,371 studies, of which 10,357 were duplicates. After screening, 83 studies were included in the meta-analysis^11–93^ (Fig. 1 and Table 1). The most evaluated models were GPT-4^3^ (54 articles) and GPT-3.5^2^ (40), while models such as GPT-4V^94^ (9), PaLM2^7^ (9), Llama 2^5^ (5), Prometheus (4), Claude 3 Opus (4)^95^, Gemini 1.5 Pro (3)^96^, GPT-4o (2)^97^, Llama 3 70B (2)^98^, Claude 3 Sonnet (2)^95^, and Perplexity (2)^99^ had less representation. Each other model, including Aya^100^, Claude 2^101^, Claude 3.5 Sonnet^102^, Clinical Camel^103^, Gemini 1.0^104^, Gemini 1.0 Pro^104^, Gemini 1.5 Flash^96^, Gemini Pro, Glass^105^, GPT-3^2^, Llama 3 8B^98^, Med-42^106^, MedAlpaca^107^, Meditron^108^, Mistral 7B^109^, Mistral Large, Mixtral8x22B^110^, Mixtral8x7B^110^, Nemotron^111^, Open Assistant^112^, WizardLM^113^, was used in only one article. Details of each model are in Supplementary Table 1 (online). The review spanned a wide range of medical specialties, with General medicine being the most common (27 articles). Other specialties like Radiology (16), Ophthalmology (11), Emergency medicine (8), Neurology (4), Dermatology (4), Otolaryngology (2), and Psychiatry (2) were represented, as well as Gastroenterology, Cardiology, Pediatrics, Urology, Endocrinology, Gynecology, Orthopedic surgery, Rheumatology, and Plastic surgery with one article each. Regarding model tasks, free text tasks were the most common, with 73 articles, followed by choice tasks at 15. For test dataset types, 59 articles involved external testing, while 25 were unknown because the training data for the generative AI models was unknown. Of the included studies, 71 were peer-reviewed, while 12 were preprints. Study characteristics are shown in Table 1 and Supplementary Table 2 (online). Seventeen studies compared the performance of generative AI models with that of physicians^36,39,43,55,72,74,75,77,78,80,81,90,93^. GPT-4 (11 articles) and GPT- 3.5 (11) were the most frequently compared with physicians, followed by GPT-4V (3), Llama 2 (2), Claude 3 Opus (1), Claude 3 Sonnet (1), Claude 3.5 Sonnet (1), Clinical Camel (1), Gemini 1.0 (1), Gemini 1.5 Flash (1), Gemini 1.5 Pro (1), Gemini Pro (1), GPT-4o (1), Llama 3 70B (1), Meditron (1), Mistral Large (1), Open Assistant (1), PaLM2 (1), Perplexity (1), Prometheus (1), and WizardLM (1).

**Table 1:** Study characteristics

Citation	First author	Model	Model task	Test type	Specialty	Comparison group	Eligible	Preprint	Overall ROB	Overall applicability
11	Ueda	GPT-4	Free text	External	Radiology	NA	313	Peer-reviewed	Low	High
12	Kanjee	GPT-4	Free text	External	General medicine	NA	70	Peer-reviewed	High	Low
13	Hirosawa	PaLM2 (Bard)	Free text	External	General medicine	NA	82	Peer-reviewed	High	Low
14	Shea	GPT-4	Free text	External	General medicine	NA	6	Peer-reviewed	High	Low
15	Chee	GPT-3.5	Free text	External	Otolaryngology	NA	7	Peer-reviewed	High	Low
16	Lyons	Prometheus (Bing), GPT-4	Free text, Choice	External	Ophthalmology	NA	44	Peer-reviewed	High	Low
17	Benoit	GPT-3.5	Free text, Choice	Unknown	General medicine	NA	45	Preprint	High	Low
18	Hirosawa	GPT-3.5, GPT-4	Free text	Unknown	General medicine	NA	52	Peer-reviewed	High	Low
19	Hirosawa	GPT-3.5	Free text	External	General medicine	NA	30	Peer-reviewed	High	Low
20	Wei	GPT-4	Choice	External	Psychiatry	NA	60	Peer-reviewed	High	Low
21	Allahqoli	GPT-3.5	Free text	Unknown	Gynecology	NA	30	Peer-reviewed	High	Low
22	Levartovsky	GPT-4	Choice	External	Gastroenterology	NA	20	Peer-reviewed	High	Low
23	Bushuven	GPT-3.5, GPT-4	Free text, Choice	External	Emergency medicine	NA	22	Peer-reviewed	High	Low
24	Knebel	GPT-3.5	Free text, Choice	External	Ophthalmology	NA	10	Peer-reviewed	High	Low
25	Pillai	GPT-3.5, GPT-4, Llama 2	Free text	Unknown	Endocrinology	Expert	20	Peer-reviewed	High	Low
26	Ito	GPT-4	Free text, Choice	Unknown	General medicine	Expert	45	Peer-reviewed	High	Low
27	Sorin	GPT-4V	Free text	External	Ophthalmology	Non-expert	40	Preprint	High	Low
28	Madadi	GPT-3.5, GPT-4	Free text	Unknown	Ophthalmology	Expert	22	Preprint	High	Low
29	Schubert	GPT-4V	Free text	External	General medicine	NA	93	Preprint	High	High
30	Kiyohara	PaLM2 (Bard), GPT-3.5, GPT-4	Choice	Unknown	Cardiology	NA	66	Preprint	High	Low
31	Sultan	GPT-3.5	Free text	External	Pediatrics	NA	30	Peer-reviewed	High	Low
32	Horiuchi	GPT-4	Free text	External	Radiology	NA	100	Peer-reviewed	Low	High
33	Stoneham	GPT-4	Free text	External	Dermatology	NA	36	Peer-reviewed	High	Low
34	Rundle	GPT-3.5	Free text	External	Dermatology	NA	39	Peer-reviewed	High	Low
35	Rojas-Carabali	GPT-3.5, GPT-4, Glass	Free text	External	Ophthalmology	NA	6	Peer-reviewed	High	Low
36	Fraser	GPT-3.5, GPT-4	Free text	Unknown	Emergency medicine	Expert	30	Peer-reviewed	High	Low
37	Krusche	GPT-4	Free text	External	Rheumatology	NA	132	Peer-reviewed	Low	Low
38	Galetta	GPT-4	Free text	External	Neurology	NA	24	Peer-reviewed	High	Low
39	Delsoz	GPT-3.5	Free text	Unknown	Ophthalmology	Non-expert	11	Peer-reviewed	High	Low
40	Hu	GPT-4	Free text	Unknown	Ophthalmology	NA	10	Peer-reviewed	High	Low
41	Abi-Rafeh	GPT-3.5	Free text	External	Plastic surgery	NA	16	Peer-reviewed	High	Low
42	Koga	PaLM2 (Bard), GPT-3.5, GPT-4	Free text	External	Neurology	NA	25	Peer-reviewed	High	Low
43	Xv	GPT-3.5	Free text	External	Urology	Non-expert	306	Peer-reviewed	Low	Low
44	Senthujan	GPT-4V	Free text	Unknown	Radiology	NA	69	Preprint	High	Low
45	Mori	GPT-4	Choice	External	Radiology	NA	151	Peer-reviewed	Low	Low
46	Mykhalko	GPT-3.5	Free text	External	General medicine	NA	50	Peer-reviewed	High	High
47	Andrade-Castellanos	GPT-3.5	Free text	External	General medicine	NA	10	Peer-reviewed	High	High
48	Daher	GPT-3.5	Free text	External	Orthopedic surgery	NA	29	Peer-reviewed	High	Low
49	Suthar	GPT-4	Free text	External	Radiology	NA	140	Peer-reviewed	Low	High
50	Nakaura	Prometheus (Bing), GPT-3.5	Free text	External	Radiology	NA	28	Peer-reviewed	High	Low
51	Berg	GPT-3.5, GPT-4	Free text	External	Emergency medicine	NA	30	Peer-reviewed	High	Low
52	Gebrael	GPT-4	Choice	External	Emergency medicine	NA	56	Peer-reviewed	High	Low
53	Ravipati	GPT-3.5	Free text	Unknown	Dermatology	NA	32	Peer-reviewed	High	Low
54	Shikino	GPT-4	Free text	External	General medicine	NA	25	Peer-reviewed	High	Low
55	Horiuchi	GPT-4, GPT-4V	Free text	External	Radiology	Non-expert, Expert	32	Peer-reviewed	High	High
56	Kumar	PaLM2 (Bard), GPT-3.5, GPT-4, Perplexity	Free text	External	Neurology	NA	20	Peer-reviewed	High	Low
57	Chiu	PaLM2 (Bard), Claude 2, GPT-4	Free text	External	General medicine	NA	104	Peer-reviewed	Low	Low
58	Kikuchi	GPT-3.5, GPT-4	Free text	External	Radiology	NA	115	Peer-reviewed	Low	High
59	Bridges	GPT-4	Free text	External	General medicine	NA	201	Peer-reviewed	High	Low
60	Shieh	GPT-4	Free text	External	General medicine	NA	63	Peer-reviewed	High	Low
61	Warrier	PaLM2 (Bard), Prometheus (Bing), GPT-3.5, GPT-4	Free text	Unknown	Otolaryngology	NA	100	Peer-reviewed	High	Low
62	Han	GPT-3.5, GPT-4, GPT-4V, Gemini 1.0 Pro, Llama 2, Med-42	Choice	External	General medicine	NA	140, 348	Peer-reviewed	Low	High
63	Milad	GPT-4	Choice	Unknown	Ophthalmology	NA	422	Peer-reviewed	High	High
64	Abdullahi	PaLM2 (Bard), GPT-3.5, GPT-4, MedAlpaca	Free text	Unknown	General medicine	NA	30	Peer-reviewed	High	High
65	Tenner	GPT-3.5	Free text	External	General medicine	NA	40	Peer-reviewed	High	Low
66	Luk	GPT-4	Free text	External	General medicine	NA	81	Peer-reviewed	High	Low
67	Savage	GPT-4	Free text	External	General medicine	NA	300	Peer-reviewed	Low	Low
68	Franc	GPT-3.5	Choice	Unknown	Emergency medicine	NA	61	Peer-reviewed	High	Low
69	Yang	GPT-3.5, GPT-4	Free text	Unknown	General medicine	NA	238	Peer-reviewed	High	Low
70	Reese	GPT-4	Free text	External	General medicine	NA	75	Preprint	High	Low
71	Olmo	Claude 3 Opus, Claude 3 Sonnet, GPT-4, Gemini 1.5 Pro, Llama 2, Llama 3 70B, Llama 3 8B, Mistral 7B, Mixtral8x22B, Mixtral8x7B	Free text	Unknown, External	General medicine	NA	200, 75	Preprint	Low	Low
72	Cesur	Claude 3 Opus, Claude 3 Sonnet, Claude 3.5 Sonnet, GPT-3.5, GPT-4, GPT-4o, Gemini 1.0, Gemini 1.5 Flash, Gemini 1.5 Pro, Llama 3 70B, Mistral Large, Perplexity	Free text	External	Radiology	Expert	80	Preprint	High	High
73	Schramm	GPT-4V	Free text	External	Neurology	NA	30	Preprint	High	Low
74	Gunes	PaLM2 (Bard), Prometheus (Bing), GPT-3.5	Free text	External	Radiology	Expert	124	Preprint	Low	High
75	Olshaker	GPT-3.5, GPT-4, Gemini Pro	Free text	Unknown	Radiology	Non-expert	60	Preprint	High	Low
76	Hirosawa	PaLM2 (Bard), GPT-4, Llama 2	Free text	External	General medicine	NA	392	Peer-reviewed	Low	Low
77	Mitsuyama	GPT-4	Free text	External	Radiology	Expert, Non-expert	150	Peer-reviewed	Low	Low
78	Yazaki	GPT-3.5, GPT-4	Choice	External	Emergency medicine	Non-expert	100	Peer-reviewed	Low	Low
79	Ghalibafan	GPT-4V	Free text	External	Ophthalmology	NA	143	Peer-reviewed	Low	Low
80	Hager	Clinical Camel, Llama 2, Meditron, Open Assistant, WizardLM	Free text	Unknown	Emergency medicine	Expert	80	Peer-reviewed	High	Low
81	Horiuchi	GPT-4, GPT-4V	Free text	External	Radiology	Expert, Non-expert	106	Peer-reviewed	Low	High
82	Ríos-Hoyo	GPT-3.5, GPT-4	Free text	External	General medicine	NA	75	Peer-reviewed	High	Low
83	Liu	Claude 3 Opus, GPT-4	Free text	External	Dermatology	NA	100	Peer-reviewed	High	Low
84	Sonoda	Claude 3 Opus, GPT-4o, Gemini 1.5 Pro	Free text	External	Radiology	NA	324	Peer-reviewed	Low	High
85	Wada	GPT-4	Free text	Unknown	Radiology	NA	751	Peer-reviewed	High	High
86	Gargari	Aya, GPT-3.5, GPT-4, Nemotron	Free text	Unknown	Psychiatry	NA	20	Peer-reviewed	High	Low
87	Mihalache	GPT-4	Free text	Unknown	Ophthalmology	NA	69	Peer-reviewed	High	High
88	Rutledge	GPT-4	Free text	Unknown	General medicine	NA	45	Peer-reviewed	High	Low
89	Ueda	GPT-4	Free text	External	General medicine	NA	62	Peer-reviewed	High	High
90	Delsoz	GPT-3.5, GPT-4	Free text	Unknown	Ophthalmology	Expert	20	Peer-reviewed	High	Low
91	Brin	GPT-4V	Free text	External	Radiology	NA	216	Peer-reviewed	Low	Low
92	Levine	GPT-3	Free text	External	General medicine	NA	48	Peer-reviewed	High	Low
93	Williams	GPT-3.5	Choice	External	Emergency medicine	Non-expert	500	Peer-reviewed	Low	Low

*ROB* risk of bias.

### Quality assessment

**Fig. 2:**
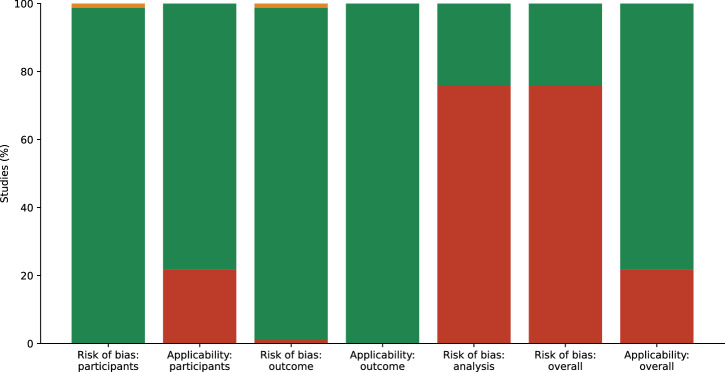
Summary of Prediction Model Study Risk of Bias Assessment Tool (PROBAST) risk of bias. Assessment for generative AI model studies included in the meta-analysis (*N* = 83). The participants and the outcome determination were predominantly at low risk of bias, but there was a high risk of bias for analysis (76%) and the overall evaluation (76%). Overall applicability and applicability for participants and outcomes are predominantly of low concern, with 22% at high concern.

Prediction Model Study Risk of Bias Assessment Tool (PROBAST) assessment led to an overall rating of 63/83 (76%) studies at high risk of bias, 20/83 (24%) studies at low risk of bias, 18/83 (22%) studies at high concern for generalizability, and 65/83 (78%) studies at low concern for generalizability^114^ (Fig. 2). The main factors of this evaluation were studies that evaluated models with a small test set and studies that cannot prove external evaluation due to the unknown training data of generative AI models. Detailed results are shown in Supplementary Table 2 (online).

### Meta-analysis

**Fig. 3:**
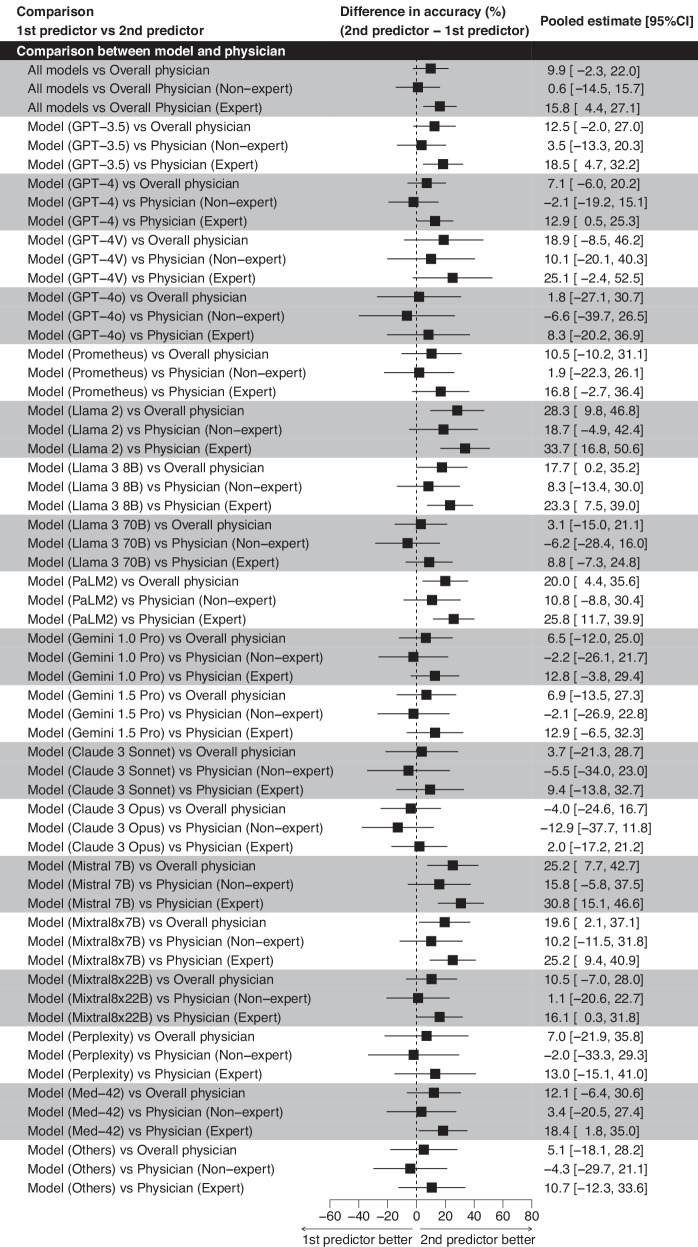
Comparison results between models and physicians. This figure demonstrates the differences in accuracy between various AI models and physicians. It specifically compares the performance of AI models against the overall accuracy of physicians, as well as against non-experts and experts separately. Each horizontal line represents the range of accuracy differences for the model compared to the physician category. The percentage values displayed on the right-hand side correspond to these mean differences, with the values in parentheses providing the 95% confidence intervals for these estimates. The dotted vertical line marks the 0% difference threshold, indicating where the model’s accuracy is exactly the same as that of the physicians. Positive values (to the right of the dotted line) suggest that the physicians outperformed the model, whereas negative values (to the left) indicate that the model was more accurate than the physicians.

The overall accuracy for generative AI models was found to be 52.1% with a 95% CI of 47.0–57.1%. The meta-analysis demonstrated no significant performance difference between generative AI models overall and physicians (physicians’ accuracy was 9.9% higher [95% CI: −2.3 to 22.0%], *p* = 0.10) and non-expert physicians (non-expert physicians’ accuracy was 0.6% higher [95% CI: −14.5 to 15.7%], *p* = 0.93), whereas generative AI models overall were significantly inferior to expert physicians (difference in accuracy: 15.8% [95% CI: 4.4–27.1%], *p* = 0.007, Fig. 3). Interestingly, several models, including GPT-4, GPT-4o, Llama3 70B, Gemini 1.0 Pro, Gemini 1.5 Pro, Claude 3 Sonnet, Claude 3 Opus, and Perplexity, demonstrated slightly higher performance compared to non-experts, although the differences were not significant. GPT-3.5, GPT-4, Llama2, Llama3 8B, PaLM2, Mistral 7B, Mixtral8x7B, Mixtral8x22B, and Med-42 were significantly inferior when compared to expert physicians, whereas GPT-4V, GPT-4o, Prometheus, Llama 3 70B, Gemini 1.0 Pro, Gemini 1.5 Pro, Claude 3 Sonnet, Claude 3 Opus, and Perplexity demonstrated no significant difference against experts.

**Fig. 4:**
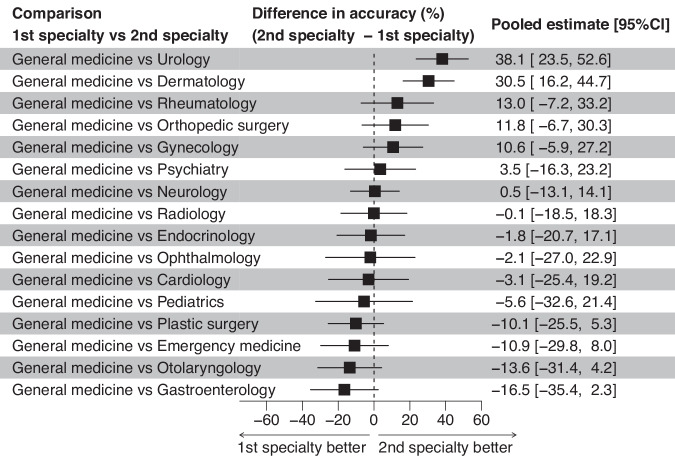
Generative AI performance among specialties. This figure demonstrates the differences in accuracy of generative AI models for specialties. Each horizontal line represents the range of accuracy differences between the specialty and General medicine. The percentage values displayed on the right-hand side correspond to these mean differences, with the values in parentheses providing the 95% confidence intervals for these estimates. The dotted vertical line marks the 0% difference threshold, indicating where the performance of generative AI models in the specialty is exactly the same as that of General medicine. Positive values (to the right of the dotted line) suggest that the model performance for the specialty was greater than that for General medicine, whereas negative values (to the left) indicate that the model performance for the specialty was less than that for General medicine.

In our meta-regression, we also found no significant difference in performance between general medicine and various specialties, except for Urology and Dermatology, where significant differences were observed (*p*-values < 0.001, Fig. 4). While medical-domain models demonstrated a slightly higher accuracy (mean difference = 2.1%, 95% CI: −28.6 to 24.3%), this difference was not statistically significant (*p* = 0.87). In the analysis of the low risk of bias subgroup, generative AI models overall demonstrated no significant performance difference compared to physicians overall (*p* = 0.069). Evaluating only studies with a low overall risk of bias showed little change compared to the full dataset results. No significant difference was observed based on the risk of bias (*p* = 0.92) or based on publication status (*p* = 0.28). We assessed publication bias by using a regression analysis to quantify funnel plot asymmetry (Supplementary Fig. 1 [online]), and it suggested a risk of publication bias (*p* = 0.045). In the heterogeneity analysis, the *R*^2^ values (amount of heterogeneity accounted for) were 45.2% for all studies and 57.1% for the studies with a low overall risk of bias, indicating moderate levels of explained variability.

## Discussion

In this systematic review and meta-analysis, we analyzed the diagnostic performance of generative AI and physicians. We initially identified 18,371 studies, ultimately including 83 in the meta-analysis. The study spanned various AI models and medical specialties, with GPT-4 being the most evaluated. Quality assessment revealed a majority of studies at high risk of bias. The meta-analysis showed a pooled accuracy of 52.1% (95% CI: 47.0–57.1%) for generative AI models. Some generative AI models showed comparable performance to non-expert physicians although no significant performance difference was found (difference in accuracy: 0.6% [95% CI: −14.5 to 15.7%], *p* = 0.93). In contrast, AI models overall were significantly inferior to expert physicians (difference in accuracy: 15.8% [95% CI: 4.4–27.1%], *p* = 0.007). Our analysis also highlighted no significant differences in effectiveness across most medical fields. To the best of our knowledge, this is the first meta-analysis of generative AI models in diagnostic tasks. This comprehensive study highlights the varied capabilities and limitations of generative AI in medical diagnostics.

The meta-analysis of generative AI models in healthcare reveals crucial insights for clinical practice. Despite the overall modest accuracy of 52% for generative AI models in medical applications, this suggests its potential utility in certain clinical scenarios. Importantly, the similar performance of generative AI models such as GPT-4, GPT-4o, Llama3 70B, Gemini 1.0 Pro, Gemini 1.5 Pro, Claude 3 Sonnet, Claude 3 Opus, and Perplexity to physicians in non-expert scenarios highlights the possibility of AI augmenting healthcare delivery in resource-limited settings or as a preliminary diagnostic tool, thereby potentially increasing accessibility and efficiency in patient care^115^. Further analysis revealed no significant difference in performance between general medicine and various specialties, except for Urology and Dermatology. These findings suggest that while there was some variation in performance across different specialties, generative AI has a wide range of capabilities. However, caution is needed when interpreting the results for Urology and Dermatology. The Urology result is based on a large single study, which may limit its generalizability. Regarding Dermatology, the superior performance may be attributed to the visual nature of the specialty, which aligns well with AI’s strengths in pattern recognition. However, it’s important to note that Dermatology involves complex clinical reasoning and patient-specific factors that go beyond visual pattern recognition. Further research is needed to elucidate the factors contributing to these specialty-specific differences in generative AI performance.

The studies comparing generative AI and physician performance also offer intriguing perspectives in the context of medical education^116^. At present, the significantly higher accuracy of expert physicians compared to AI models overall emphasizes the irreplaceable value of human judgment and experience in medical decision-making. However, the comparable performance of current generative AI models to physicians in non-expert settings reveals an opportunity for integrating AI into medical training. This could include using AI as a teaching aid for medical students and residents, especially in simulating non-expert scenarios where AI’s performance is nearly equivalent to that of healthcare professionals^117^. Such integration could enhance learning experiences, offering diverse clinical case studies and facilitating self-assessment and feedback. Additionally, the narrower performance gap between some generative AI models and physicians, even in expert settings, suggests that AI could be used to supplement advanced medical education, helping to identify areas for improvement and providing supporting information. This approach could foster a more dynamic and adaptive learning environment, preparing future medical professionals for an increasingly digital healthcare landscape.

To examine the impact of the overall risk of bias, we conducted a subgroup analysis of studies with a low overall risk of bias. The result of studies with a low overall risk of bias showed little change compared to that of the full dataset. This result suggests that the high proportion of studies with high overall risk of bias does not substantially affect our study’s findings or generalizability. While many generative AI models do not disclose details of their training data, the transparency of training data and its collection period is paramount. Without this transparency, it is impossible to determine whether the test dataset is an external dataset or not and this can lead to bias. Transparency ensures an understanding of the model’s knowledge, context, and limitations, aids in identifying potential biases, and facilitates independent replication and validation, which are fundamental to scientific integrity. As generative AI continues to evolve, fostering a culture of rigorous transparency is essential to ensure its safe, effective, and equitable application in clinical settings^118^, ultimately enhancing the quality of healthcare delivery and medical education.

The methodology of this study, while comprehensive, has limitations. The generalizability of our findings warrants careful consideration. Our heterogeneity analysis revealed moderate levels of explained variability, suggesting that while our meta-regression model accounts for a substantial portion of the differences between studies, other factors not captured in our analysis may influence generative AI performance. Furthermore, the lack of demographic information in many included studies limits our ability to assess the generalizability of these findings across diverse populations and geographic regions. The performance of generative AI may vary considerably depending on the demographic characteristics and healthcare contexts represented in their training data. Although our meta-analysis shows no significant difference between the pooled accuracy of models and that of physicians, recent research demonstrates that generative AI may perform significantly worse than physicians in more complex scenarios where models are provided with detailed information from electronic health records^80^. This suggests that generative AI model performance may degrade in more complex, real-world scenarios. Future research should prioritize the inclusion of diverse patient populations and cases that reflect more complex, real-world scenarios to better understand the generalizability of generative AI performance across different populations and clinical settings. Additionally, investigating the intersecting impact of physicians using generative AI models clinically, such as changes in performance, would be valuable.

In conclusion, this meta-analysis provides a nuanced understanding of the capabilities and limitations of generative AI in medical diagnostics. Generative AI models, particularly advanced iterations like GPT-4, GPT-4o, Llama3 70B, Gemini 1.0 Pro, Gemini 1.5 Pro, Claude 3 Sonnet, Claude 3 Opus, and Perplexity, have shown progressive improvements and hold promise for assisting in diagnosis, though their effectiveness varies by model. With an overall moderate accuracy of 52%, generative AI models are not yet reliable substitutes for expert physicians but may serve as valuable aids in non-expert scenarios and as educational tools for medical trainees. The findings also underscore the need for continued advancements and specialization in model development, as well as rigorous, externally validated research to overcome the prevalent high risk of bias and ensure generative AIs’ effective integration into clinical practice. As the field evolves, continuous learning and adaptation for both generative AI models and medical professionals are imperative, alongside a commitment to transparency and stringent research standards. This approach will be crucial in harnessing the potential of generative AI models to enhance healthcare delivery and medical education while safeguarding against their limitations and biases.

## Methods

### Protocol and registration

This systematic review was prospectively registered with PROSPERO (CRD42023494733). Our study adhered to the relevant sections of guidelines from the Preferred Reporting Items for a Systematic Review and Meta-analysis (PRISMA) of Diagnostic Test Accuracy Studies (Supplementary Table 4)^119,120^. All stages of the review (title and abstract screening, full-text screening, data extraction, and assessment of bias) were performed in duplicate by two independent reviewers (H.Takita and D.U.), and disagreements were resolved by discussion with a third independent reviewer (H.Tatekawa).

### Search strategy and study selection

A search was performed to identify studies that validate a generative AI model for diagnostic tasks. A search strategy was developed, including variations of the terms *generative AI* and *diagnosis*. The search strategy was as follows: articles in English that included the words “large language model,” “LLM,” “generative artificial intelligence,” “generative AI,” “generative pre-trained transformers,”^1^ “GPT,” “Bing,” “Prometheus,” “Bard,” “PaLM,”^6,7^ “Pathways Language Model,” “LaMDA,”^8^ “Language Model for Dialogue Applications,” “Llama,”^4,5^ or “Large Language Model Meta AI” and also “diagnosis,” “diagnostic,” “quiz,” “examination,” or “vignette” were included. We searched the following electronic databases for literature from June 2018 through June 2024: Medline, Scopus, Web of Science, Cochrane Central, and MedRxiv. June 2018 represents when the first generative AI model was published^1^. We included all articles that fulfilled the following inclusion criteria: primary research studies that validate a generative AI for diagnosis. We applied the following exclusion criteria to our search: review articles, case reports, comments, editorials, and retracted articles.

### Data extraction

Titles and abstracts were screened before full-text screening. Data was extracted using a predefined data extraction sheet. A count of excluded studies, including the reason for exclusion, was recorded in a PRISMA flow diagram^120^. We extracted information from each study including the first author, model with its version, model task, test dataset type (internal, external, or unknown)^114^, medical specialty, accuracy, sample size, and publication status (preprint or peer-reviewed) for the meta-analysis of generative AI performance. We defined three test types based on the relationship between the model’s training and test data^121^. We defined internal testing as when the test data was derived from the same source or distribution as the training data but was properly separated from the training set through standard practices such as cross-validation or random splitting. We defined external testing as when the test data was collected after the training data cutoff, or the model was tested on private data. We defined unknown testing as when the test data was collected before the training data cutoff, and the data was publicly available. This is because companies developing these models have not disclosed their complete training datasets. In addition to this, when both the model and the physician’s diagnostic performance were presented in the same paper, we extracted both for meta-analysis. We also considered the type of physician involved in relevant studies. We classified physicians as non-experts if they were trainees or residents. In contrast, those beyond this stage in their career were categorized as experts. When a single model used multiple prompts and individual performances were available in one article, we took the average of them.

### Quality assessment

We used PROBAST to assess papers for bias and applicability^114^. This tool uses signaling questions in four domains (participants, predictors, outcomes, and analysis) to provide both an overall and a granular assessment. We did not include some PROBAST signaling questions because they are not relevant to generative AI models. Details of modifications made to PROBAST are in Supplementary Table 3 (online).

### Statistical analysis

We calculated the pooled accuracy of diagnosis brought by generative AI models and physicians based on the previously reported studies. The pooled diagnosis accuracies were compared between all AI models and physicians overall using the multivariable random-effect meta-regression model with adjustment for medical specialty, task of models, type of test dataset, level of bias, and publication status. We compared AI models overall with physicians overall, expert physicians, and non-expert physicians. Additionally, we compared each AI model with physicians overall and each AI model with each physician’s experience level (expert or non-expert). Furthermore, we assessed the variation of generative AI model accuracy across specialties. For fitting the meta-regression models, a restricted maximum likelihood estimator was utilized with the “metafor” package in R. To explore variation between knowledge domains, we performed a subgroup analysis comparing medical-domain models with non-medical-domain models. To assess the impact of the overall risk of bias, we conducted a subgroup analysis limited to studies with a low overall risk of bias. To assess the impact of publication bias on the comparison of the diagnosis performance between the AI models and the physicians, we used a funnel plot and Egger’s regression test. Additionally, to assess the impact of heterogeneity, we conducted heterogeneity analyses in both the full dataset and the subgroup that had a low overall risk of bias. All statistical analyses were conducted using R version 4.4.0.

## Supplementary information

Supplementary Information

## Data Availability

The corresponding author had full access to all data in the study and final responsibility for the decision to submit the report for publication. The data used and analyzed during the current study are available from the corresponding author upon reasonable request.

## Code availability

Study protocol and metadata are available from Dr. Ueda (e-mail, ai.labo.ocu@gmail.com).
