# Supplementary material for: A systematic review and meta-analysis of diagnostic performance comparison between generative AI and physicians
Source: NPJ Digit Med. 2025 Mar 22;8:175. doi: 10.1038/s41746-025-01543-z (PMC11929846; doi:10.1038/s41746-025-01543-z)
Supplement: Supplementary file 1 — Supplementary Information [file 41746_2025_1543_MOESM1_ESM.pdf]

A Systematic Review and Meta-analysis of Diagnostic Performance Comparison between Generative AI and Physicians

Hirotaka Takita, Daijiro Kabata, Shannon L Walston, Hiroyuki Tatekawa, Kenichi Saito, Yasushi Tsujimoto, Yukio Miki, Daiju Ueda

Table of Contents:

Section S1: Supplementary Figure

Supplementary Figure 1: Funnel plot

Section S2: Supplementary Tables

Supplementary Table 1: Models' description
Supplementary Table 2: Detailed study characteristics
Supplementary Table 3: PROBAST modifications
Supplementary Table 4: PRISMA 2020 checklist

Section S1: Supplementary Figure
Supplementary Figure 1: Funnel plot

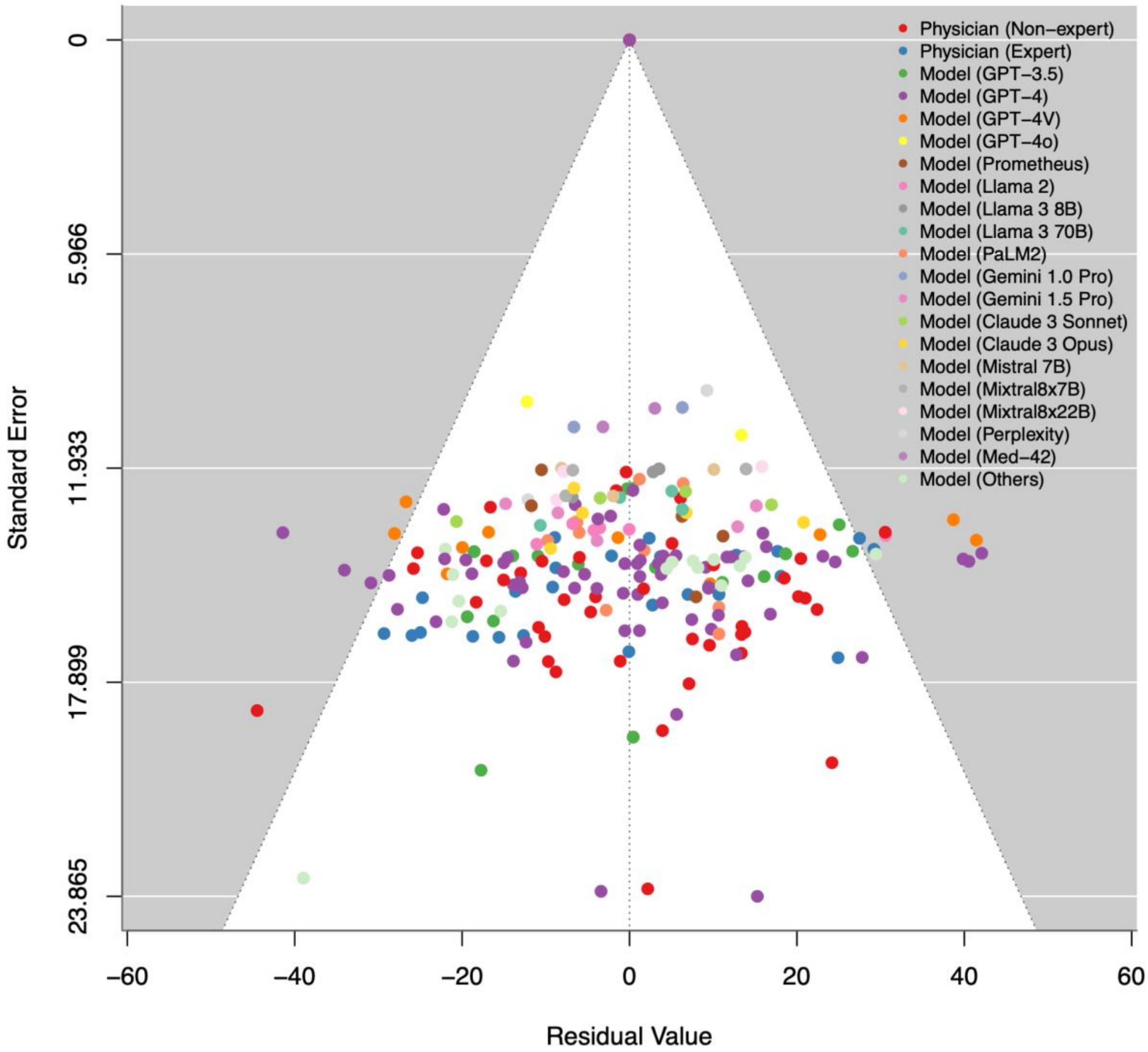

The funnel plot illustrates the distribution of the residuals of the fitted values corresponding to their standard errors in the meta-regression. The Egger test results z-value = -1.9982 and p = 0.045, which indicates the possible presence of publication bias.

Section S2: Supplementary Tables

Supplementary Table 1: Models' description

**Model**	**Description**
Aya	Open-source large language model (Cohere for AI, Toronto, Canada)
Claude 2, Claude 3 Opus, Claude 3 Sonnet, Claude 3.5 Sonnet	Closed-source large language models (Anthropic, San Francisco, CA)
Clinical Camel	Fine-tuned medical-domain version of Llama
Gemini 1.0 Pro, Gemini 1.5 Pro, Gemini 1.5 Flash	Subsequent large language models to Bard (Google, Menlo Park, CA)
Glass	Closed-source and medical-domain large language model (Glass Health, San Francisco, CA)
GPT-3, GPT-3.5, GPT-4, GPT-4V, GPT-4o	Closed-source large language models (Open AI, San Francisco, CA)
Llama 2, Llama 3 8B, Llama 3 70B	Open-source large language models (Meta, Menlo Park, CA)
Med-42	Fine-tuned medical-domain version of Llama
MedAlpaca	Fine-tuned medical-domain version of Llama
Meditron	Fine-tuned medical-domain version of Llama
Mistral 7B, Mistral Large	Closed-source large language models (Mistral AI, Paris, France)
Mixtral8x22B, Mixtral8x7B	Open-source large language models (Mistral AI, Paris, France)
Nemotron	Open-source large language model (NVIDIA, Santa Clara, CA)
Open Assistant	Open-source large language model (Large-scale Artificial Intelligence Open Network)
PaLM2	Closed-source large language model implemented in Bard (Google, Menlo Park, CA)
Perplexity	Closed-source large language model with search engines (Perplexity AI, San Francisco, CA)
Prometheus	Closed-source large language model implemented in Bing (Microsoft, Redmond, WA)
WizardLM	Fine-tuned version of Llama

Supplementary Table 2: Detailed study characteristics

Citation	Publication Year	First Author	Model	Version	Database	Reference standard	ROB of Participants	Applicability of participants	ROB of Outcome	Applicability of outcome	ROB of analysis
11	2023	Ueda	GPT-4	Not written	The quiz "Diagnosis Please" in the Radiology	Answer	Low	High	Low	Low	Low
12	2023	Kanjee	GPT-4	Not written	Clinical vignettes from case reports in the NEJM	Expert consensus	Low	Low	Low	Low	High
13	2023	Hirosawa	PaLM2 (Bard)	Not written	Clinical vignettes representing various common complaints	Expert consensus	Low	Low	Low	Low	High
14	2023	Shea	GPT-4	Not written	Clinical vignettes about patients who had delay of definitive diagnosis longer than 1 month	Expert consensus	Low	Low	Low	Low	High
15	2023	Chee	GPT-3.5	Not written	Hypothetical clinical vignettes representing vertigo	Expert consensus	Unclear	Low	High	Low	High
16	2023	Lyons	Prometheus (Bing), GPT-4	Not written	Hypothetical clinical vignettes representing common ophthalmic complaints	Expert consensus	Low	Low	Low	Low	High
17	2023	Benoit	GPT-3.5	09-Jan-23	Clinical vignettes representing various common complaints	Expert consensus	Low	Low	Unclear	Low	High
18	2023	Hirosawa	GPT-3.5, GPT-4	March 14, 2023, March 25, 2023	Clinical vignettes about general internal medicine	Expert consensus	Low	Low	Low	Low	High
19	2023	Hirosawa	GPT-3.5	15-Dec-23	Clinical vignettes about general internal medicine	Expert consensus	Low	Low	Low	Low	High
20	2023	Wei	GPT-4	Not written	Clinical vignettes about psychiatry	Expert consensus	Low	Low	Low	Low	High

21	2023	Allahqoli	GPT-3.5	Not written	Clinical vignettes about obstetrics and gynecology	Expert consensus	Low	Low	Low	Low	High
22	2023	Levartovsky	GPT-4	Not written	Clinical vignettes about patients with ulcerative colitis	Expert consensus	Low	Low	Low	Low	High
23	2023	Bushuven	GPT-3.5, GPT-4	23-Mar-23	Clinical vignettes about emergency medicine	Expert consensus	Low	Low	Low	Low	High
24	2023	Knebel	GPT-3.5	14-Mar-23	Hypothetical clinical vignettes representing acute ocular symptoms	Expert consensus	Low	Low	Low	Low	High
25	2023	Pillai	GPT-3.5, GPT-4, Llama 2	Not written	Clinical vignettes about FMF and DIRA	Expert consensus	Low	Low	Low	Low	High
26	2023	Ito	GPT-4	14-Mar-23	Clinical vignettes representing various common complaints	Expert consensus	Low	Low	Low	Low	High
27	2023	Sorin	GPT-4V	Not written	Clinical vignettes about patients with ocular symptoms	Expert consensus	Low	Low	Low	Low	High
28	2023	Madadi	GPT-3.5, GPT-4	Not written	Clinical vignettes about ophthalmology	Expert consensus	Low	Low	Low	Low	High
29	2023	Schubert	GPT-4V	Not written	The quiz “Image Challenge” in the NEJM	Answer	Low	High	Low	Low	High
30	2023	Kiyohara	PaLM2 (Bard), GPT-3.5, GPT-4	Not written	Clinical vignettes about vasospastic angina and acute coronary syndrome	Expert consensus	Low	Low	Low	Low	High
31	2023	Sultan	GPT-3.5	Not written	Clinical vignettes about pediatric patients with cancer predisposition syndromes	Expert consensus	Low	Low	Low	Low	High
32	2023	Horiuchi	GPT-4	03-Aug-23	The quiz “Case of the Week” in the AJNR	Answer	Low	High	Low	Low	Low

33	2023	Stoneham	GPT-4	Not written	Clinical vignettes about patients with cutaneous symptoms	Expert consensus	Low	Low	Low	Low	High
34	2023	Rundle	GPT-3.5	24-May-23	Hypothetical clinical vignettes representing cutaneous tumors	Expert consensus	Low	Low	Low	Low	High
35	2023	Rojas-Carabali	GPT-3.5, GPT-4, Glass	Not written	Clinical vignettes about patients with uveritis	Expert consensus	Low	Low	Low	Low	High
36	2023	Fraser	GPT-3.5, GPT-4	Not written	Clinical vignettes about emergency medicine	Expert consensus	Low	Low	Low	Low	High
37	2023	Krusche	GPT-4	Not written	Clinical vignettes about rheumatology	Expert consensus	Low	Low	Low	Low	Low
38	2023	Galetta	GPT-4	Not written	Clinical vignettes about neurology	Expert consensus	Low	Low	Low	Low	High
39	2023	Delsoz	GPT-3.5	Not written	Clinical vignettes about ophthalmology	Expert consensus	Low	Low	Low	Low	High
40	2023	Hu	GPT-4	Not written	Clinical vignettes about ophthalmology	Expert consensus	Low	Low	Low	Low	High
41	2023	Abi-Rafeh	GPT-3.5	Not written	Hypothetical clinical vignettes about Plastic surgery	Expert consensus	Low	Low	Low	Low	High
42	2023	Koga	PaLM2 (Bard), GPT-3.5, GPT-4	Not written	Clinical vignettes about neuropathology	Expert consensus	Low	Low	Low	Low	High
43	2023	Xv	GPT-3.5	Not written	Clinical vignettes about urology	Expert consensus	Low	Low	Low	Low	Low
44	2023	Senthujan	GPT-4V	Not written	Clinical vignettes about various medical images	Expert consensus	Low	Low	Low	Low	High
45	2023	Mori	GPT-4	Not written	Clinical vignettes about imaging findings of SAPHO syndrome	Expert consensus	Low	Low	Low	Low	Low

46	2023	Mykhalko	GPT-3.5	Not written	The quiz “Case Challenges” from the Medscape website	Answer	Low	High	Low	Low	High
47	2023	Andrade-Castellanos	GPT-3.5	Not written	The quiz “Test yourself” from the American College of Physicians website	Answer	Low	High	Low	Low	High
48	2023	Daher	GPT-3.5	Not written	Clinical vignettes about patients with joint symptoms	Expert consensus	Low	Low	Low	Low	High
49	2023	Suthar	GPT-4	20-Jul-23	The quiz “Case of the Month” in the AJNR	Answer	Low	High	Low	Low	Low
50	2023	Nakaura	Prometheus (Bing), GPT-3.5	Not written, June 13, 2023	Clinical vignettes about various imaging findings	Expert consensus	Low	Low	Low	Low	High
51	2023	Berg	GPT-3.5, GPT-4	Not written	Clinical vignettes about emergency medicine	Expert consensus	Low	Low	Low	Low	High
52	2023	Gebrael	GPT-4	Not written	Clinical vignettes about patients with prostate cancer in emergency rooms	Expert consensus	Low	Low	Low	Low	High
53	2023	Ravipati	GPT-3.5	Not written	Clinical vignettes about dermatology	Expert consensus	Low	Low	Low	Low	High
54	2024	Shikino	GPT-4	12-Mar-24	Clinical vignettes in the Journal of Generalist Medicine	Answer	Low	Low	Low	Low	High
55	2024	Horiuchi	GPT-4, GPT-4V	Feb 13, 2024, Feb 13, 2025	The quiz "Freiburg Neuropathology Case Conference" in the Clinical Neuroradiology	Answer	Low	High	Low	Low	High
56	2024	Kumar	PaLM2 (Bard), GPT-3.5, GPT-4, Perplexity	Sep, 2023	Hypothetical clinical vignettes about neurosurgery	Answer	Low	Low	Low	Low	High
57	2024	Chiu	PaLM2 (Bard), Claude 2, GPT-4	Not written	Clinical vignettes from case reports in the NEJM	Answer	Low	Low	Low	Low	Low

58	2024	Kikuchi	GPT-3.5, GPT-4	gpt-3.5-turbo, Feb 20, 2024	The quiz “Case of the Week” in the AJNR	Answer	Low	High	Low	Low	Low
59	2024	Bridges	GPT-4	Not written	Clinical vignettes from case reports in the NEJM and a previous paper	Answer	Low	Low	Low	Low	High
60	2024	Shieh	GPT-4	Not written	Clinical vignettes about general internal medicine	Answer	Low	Low	Low	Low	High
61	2024	Warrier	PaLM2 (Bard), Prometheus (Bing), GPT-3.5, GPT-4	June, 2023, Jan, 2024	Clinical vignettes about Otolaryngology	Answer	Low	Low	Low	Low	High
62	2024	Han	GPT-3.5, GPT-4, GPT-4V, Gemini 1.0 Pro, Llama 2, Med-42	Not written	The quiz “Image Challenge” in the NEJM and the quiz "Image of the Month" in the JAMA	Answer	Low	High	Low	Low	Low
63	2024	Milad	GPT-4	July, 2023	The quiz from JAMA Ophthalmology’s Clinical Challenges	Answer	Low	High	Low	Low	High
64	2024	Abdullahi	PaLM2 (Bard), GPT-3.5, GPT-4, MedAlpaca	July 6, 2023, July 4, 2023, MedAlpaca 7b	The quiz “Image Challenge” in the NEJM	Answer	Low	High	Low	Low	High
65	2024	Tenner	GPT-3.5	Not written	Clinical vignettes from case reports in the NEJM	Answer	Low	Low	Low	Low	High
66	2024	Luk	GPT-4	Not written	Clinical vignettes from case reports in the NEJM	Answer	Low	Low	Low	Low	High
67	2024	Savage	GPT-4	Not written	Clinical vignettes from case reports in the NEJM	Answer	Low	Low	Low	Low	Low

68	2024	Franc	GPT-3.5	gpt-3.5-turbo	Clinical vignettes about emergency medicine	Answer	Low	Low	Low	Low	High
69	2024	Yang	GPT-3.5, GPT-4	14-Mar-23	Clinical vignettes about general internal medicine	Answer	Low	Low	Low	Low	High
70	2024	Reese	GPT-4	Not written	Clinical vignettes from case reports in the NEJM	Answer	Low	Low	Low	Low	High
71	2024	Olmo	Claude 3 Opus, Claude 3 Sonnet, GPT-4, Gemini 1.5 Pro, Llama 2, Llama 3 70B, Llama 3 8B, Mistral 7B, Mixtral8x22B, Mixtral8x7B	Not written, 0613_ver, Turbo 1106, Turbo 0409, 7B, 70B, 8B	Hypothetical and real clinical vignettes about rare disease	Answer	Low	Low	Low	Low	Low
72	2024	Cesur	Claude 3 Opus, Claude 3 Sonnet, Claude 3.5 Sonnet, GPT-3.5, GPT-4, GPT-4o, Gemini 1.0, Gemini 1.5 Flash, Gemini 1.5 Pro, Llama 3 70B, Mistral Large, Perplexity	May-24	The quiz “Cardiac Case of the Month” in The Society of Thoracic Radiology	Answer	Low	High	Low	Low	High
73	2024	Schramm	GPT-4V	Not written	Clinical vignettes about neurology	Answer	Low	Low	Low	Low	High
74	2024	Gunes	PaLM2 (Bard), Prometheus (Bing), GPT-3.5	Jan, 2024	The quiz “Case of the Month” in The Society of Thoracic Radiology	Answer	Low	High	Low	Low	Low
75	2024	Olshaker	GPT-3.5, GPT-4, Gemini Pro	Not written	Clinical vignettes about imaging findings of multisystemic syndromes	Answer	Low	Low	Low	Low	High

76	2024	Hirosawa	PaLM2 (Bard), GPT-4, Llama 2	Not written, March 24, 2023, 70B	Clinical vignettes about general internal medicine	Answer	Low	Low	Low	Low	Low
77	2024	Mitsuyama	GPT-4	24-May-23	Clinical vignettes about imaging findings of brain tumors	Answer	Low	Low	Low	Low	Low
78	2024	Yazaki	GPT-3.5, GPT-4	GPT-3.5-turbo 0613, gpt-4-1106	Hypothetical clinical vignettes about emergency medicine	Answer	Low	Low	Low	Low	Low
79	2024	Ghalibafan	GPT-4V	gpt-4-1106	Clinical vignettes about Ophthalmology	Answer	Low	Low	Low	Low	Low
80	2024	Hager	Clinical Camel, Llama 2, Meditron, Open Assistant, WizardLM	70B	Clinical vignettes from MIMIC-IV dataset	Answer	Low	Low	Low	Low	High
81	2024	Horiuchi	GPT-4, GPT-4V	25-Sep-23	The quiz “Test yourself” in the Skeletal Radiology	Answer	Low	High	Low	Low	Low
82	2024	Ríos-Hoyo	GPT-3.5, GPT-4	gpt-3.5-turbo, gpt-4	Clinical vignettes from case reports in the NEJM	Answer	Low	Low	Low	Low	High
83	2024	Liu	Claude 3 Opus, GPT-4	Not written	The dermoscopic images from the International Skin Imaging Collaboration archive	Answer	Low	Low	Low	Low	High
84	2024	Sonoda	Claude 3 Opus, GPT-4o, Gemini 1.5 Pro	claude-3-opus-20240229, gpt-4o-2024-05-13, May 18, 2024	The quiz “Diagnosis Please” in the Radiology	Answer	Low	High	Low	Low	Low
85	2024	Wada	GPT-4	Not written	The quiz “Case of the Week” in the AJNR	Answer	Low	High	Low	Low	High

86	2024	Gargari	Aya, GPT-3.5, GPT-4, Nemotron	Aya-101, Not written, Nemotron-3–8B-CHAT-4K-RLHF	Clinical vignettes from "DSM-5 Clinical Cases"	Answer	Low	Low	Low	Low	High
87	2024	Mihalache	GPT-4	06-Dec-23	The retinal images from the medical platform of the University of Toronto	Answer	Low	High	Low	Low	High
88	2024	Rutledge	GPT-4	Jan, 2024	Clinical vignettes from general internal medicine	Answer	Low	Low	Low	Low	High
89	2024	Ueda	GPT-4	23-Mar-23	The quiz "Image Challenge" in the NEJM	Answer	Low	High	Low	Low	High
90	2024	Delsoz	GPT-3.5, GPT-4	Not written	Clinical vignettes about Ophthalmology	Answer	Low	Low	Low	Low	High
91	2024	Brin	GPT-4V	Not written	Clinical vignettes about imaging findings	Answer	Low	Low	Low	Low	Low
92	2024	Levine	GPT-3	July, 2022	Clinical vignettes about general internal medicine	Answer	Low	Low	Low	Low	High
93	2024	Williams	GPT-3.5	gpt-3.5-turbo-0301	Clinical vignettes about emergency medicine	Answer	Low	Low	Low	Low	Low

ROB risk of bias

Supplementary Table 3: PROBAST modifications

PROBAST Items	Modifications
Domain 1: Participants	No changes (Refer to participant data for diagnosis)
Domain 2: Predictors	N/A–removed from scoring
Domain 3: Outcome	Items 3.3, 3.5, and 3.6 N/A
Domain 4: Analysis	Items 4.5, 4.6, and 4.9 N/A
Domain 5: Overall	No changes

Supplementary Table 4: PRISMA 2020 checklist

Section and Topic	Item #	Checklist item	Location where item is reported
**TITLE**			
Title	1	Identify the report as a systematic review.	Title
**ABSTRACT**			
Abstract	2	See the PRISMA 2020 for Abstracts checklist.	
**INTRODUCTION**			
Rationale	3	Describe the rationale for the review in the context of existing knowledge.	Third paragraph
Objectives	4	Provide an explicit statement of the objective(s) or question(s) the review addresses.	Fourth paragraph
**METHODS**			
Eligibility criteria	5	Specify the inclusion and exclusion criteria for the review and how studies were grouped for the syntheses.	Search Strategy and Study Selection
Information sources	6	Specify all databases, registers, websites, organisations, reference lists and other sources searched or consulted to identify studies. Specify the date when each source was last searched or consulted.	Search Strategy and Study Selection
Search strategy	7	Present the full search strategies for all databases, registers and websites, including any filters and limits used.	Search Strategy and Study Selection
Selection process	8	Specify the methods used to decide whether a study met the inclusion criteria of the review, including how many reviewers screened each record and each report retrieved, whether they worked independently, and if applicable, details of automation tools used in the process.	Protocol and Registration, Search Strategy and Study Selection
Data collection process	9	Specify the methods used to collect data from reports, including how many reviewers collected data from each report, whether they worked independently, any processes for obtaining or confirming data from study investigators, and if applicable, details of automation tools used in the process.	Protocol and Registration, Search Strategy and Study Selection
Data items	10a	List and define all outcomes for which data were sought. Specify whether all results that were compatible with each outcome domain in each study were sought (e.g. for all measures, time points, analyses), and if not, the methods used to decide which results to collect.	Data Extraction, Table1, Supplementary Table 2
	10b	List and define all other variables for which data were sought (e.g. participant and intervention characteristics, funding sources). Describe any assumptions made about any missing or unclear information.	Data Extraction, Table1, Supplementary Table 2

Study risk of bias assessment	11	Specify the methods used to assess risk of bias in the included studies, including details of the tool(s) used, how many reviewers assessed each study and whether they worked independently, and if applicable, details of automation tools used in the process.	Quality Assessment
Effect measures	12	Specify for each outcome the effect measure(s) (e.g. risk ratio, mean difference) used in the synthesis or presentation of results.	Statistical Analysis
Synthesis methods	13a	Describe the processes used to decide which studies were eligible for each synthesis (e.g. tabulating the study intervention characteristics and comparing against the planned groups for each synthesis (item #5)).	Statistical Analysis
	13b	Describe any methods required to prepare the data for presentation or synthesis, such as handling of missing summary statistics, or data conversions.	Statistical Analysis
	13c	Describe any methods used to tabulate or visually display results of individual studies and syntheses.	Statistical Analysis
	13d	Describe any methods used to synthesize results and provide a rationale for the choice(s). If meta-analysis was performed, describe the model(s), method(s) to identify the presence and extent of statistical heterogeneity, and software package(s) used.	Statistical Analysis
	13e	Describe any methods used to explore possible causes of heterogeneity among study results (e.g. subgroup analysis, meta-regression).	Statistical Analysis
	13f	Describe any sensitivity analyses conducted to assess robustness of the synthesized results.	Statistical Analysis
Reporting bias assessment	14	Describe any methods used to assess risk of bias due to missing results in a synthesis (arising from reporting biases).	Quality Assessment, Statistical Analysis
Certainty assessment	15	Describe any methods used to assess certainty (or confidence) in the body of evidence for an outcome.	Quality Assessment, Statistical Analysis
**RESULTS**			
Study selection	16a	Describe the results of the search and selection process, from the number of records identified in the search to the number of studies included in the review, ideally using a flow diagram.	Study Selection and Characteristics, Figure 1
	16b	Cite studies that might appear to meet the inclusion criteria, but which were excluded, and explain why they were excluded.	Figure 1
Study characteristics	17	Cite each included study and present its characteristics.	Study Selection and Characteristics
Risk of bias in studies	18	Present assessments of risk of bias for each included study.	Quality Assessment
Results of individual studies	19	For all outcomes, present, for each study: (a) summary statistics for each group (where appropriate) and (b) an effect estimate and its precision (e.g. confidence/credible interval), ideally using structured tables or plots.	Meta-analysis
Results of syntheses	20a	For each synthesis, briefly summarise the characteristics and risk of bias among contributing studies.	Meta-analysis

	20b	Present results of all statistical syntheses conducted. If meta-analysis was done, present for each the summary estimate and its precision (e.g. confidence/credible interval) and measures of statistical heterogeneity. If comparing groups, describe the direction of the effect.	Meta-analysis
	20c	Present results of all investigations of possible causes of heterogeneity among study results.	Meta-analysis
	20d	Present results of all sensitivity analyses conducted to assess the robustness of the synthesized results.	Meta-analysis
Reporting biases	21	Present assessments of risk of bias due to missing results (arising from reporting biases) for each synthesis assessed.	Meta-analysis
Certainty of evidence	22	Present assessments of certainty (or confidence) in the body of evidence for each outcome assessed.	Meta-analysis
**DISCUSSION**			
Discussion	23a	Provide a general interpretation of the results in the context of other evidence.	Fifth paragraph
	23b	Discuss any limitations of the evidence included in the review.	Fifth paragraph
	23c	Discuss any limitations of the review processes used.	Fifth paragraph
	23d	Discuss implications of the results for practice, policy, and future research.	Second and third paragraph
**OTHER INFORMATION**			
Registration and protocol	24a	Provide registration information for the review, including register name and registration number, or state that the review was not registered.	Protocol and Registration in the Methods
	24b	Indicate where the review protocol can be accessed, or state that a protocol was not prepared.	Protocol and Registration in the Methods
	24c	Describe and explain any amendments to information provided at registration or in the protocol.	Protocol and Registration in the Methods
Support	25	Describe sources of financial or non-financial support for the review, and the role of the funders or sponsors in the review.	Acknowledgements
Competing interests	26	Declare any competing interests of review authors.	Competing Interests
Availability of data, code and other materials	27	Report which of the following are publicly available and where they can be found: template data collection forms; data extracted from included studies; data used for all analyses; analytic code; any other materials used in the review.	Data availability, Code availability

From: Page MJ, McKenzie JE, Bossuyt PM, Boutron I, Hoffmann TC, Mulrow CD, et al. The PRISMA 2020 statement: an updated guideline for reporting systematic reviews. BMJ 2021;372:n71. doi: 10.1136/bmj.n71
